# A model for hydrophobic protrusions on peripheral membrane proteins

**DOI:** 10.1371/journal.pcbi.1006325

**Published:** 2018-07-26

**Authors:** Edvin Fuglebakk, Nathalie Reuter

**Affiliations:** 1 Computational Biology Unit, University of Bergen, Bergen, Norway; 2 Department of Molecular Biology, University of Bergen, Bergen, Norway; 3 Department of Chemistry, University of Bergen, Bergen, Norway; University of Oxford, UNITED KINGDOM

## Abstract

With remarkable spatial and temporal specificities, peripheral membrane proteins bind to biological membranes. They do this without compromising solubility of the protein, and their binding sites are not easily distinguished. Prototypical peripheral membrane binding sites display a combination of patches of basic and hydrophobic amino acids that are also frequently present on other protein surfaces. The purpose of this contribution is to identify simple but essential components for membrane binding, through structural criteria that distinguish exposed hydrophobes at membrane binding sites from those that are frequently found on any protein surface. We formulate the concepts of *protruding hydrophobes* and *co-insertability* and have analysed more than 300 families of proteins that are classified as peripheral membrane binders. We find that this structural motif strongly discriminates the surfaces of membrane-binding and non-binding proteins. Our model constitutes a novel formulation of a structural pattern for membrane recognition and emphasizes the importance of subtle structural properties of hydrophobic membrane binding sites.

## Author summary

Peripheral membrane proteins bind cellular membranes transiently, and are otherwise soluble proteins. As the interaction between proteins and membranes happens at cellular interfaces they are naturally involved in important interfacial processes such as recognition, signaling and trafficking. Commonly their binding sites are also soluble, and their binding mechanisms poorly understood. This complicates the elaboration of conceptual and quantitative models for peripheral membrane binding and makes binding site prediction difficult. It is therefore of great interest to discover traits that are common between these binding sites and that distinguishes them from other protein surfaces. In this work we identify simple and general structural features that facilitate membrane recognition by soluble proteins. We show that these motifs are highly over-represented on peripheral membrane proteins.

## Introduction

Biological membranes are ancient and crucial components in the organisation of life. Not only do they define the boundaries of cells and organelles, but they are central to a myriad protein-protein and protein-lipid interactions instrumental in numerous pathways [1–5]. Besides the embedded transmembrane proteins and receptors, a number of soluble proteins interact transiently with the surface of cellular and organellar membranes achieving remarkable spatial and temporal specificities. These proteins (or domains) are referred to as peripheral proteins (or domains) and their membrane-binding site as interfacial binding site or IBS. Peripheral proteins may bind membranes via lipid-binding *domains* which are independently folded modules forming an integral part of the overall protein; C2-domains and FYVE-domains are examples of such domains [6, 7]. Many lipid-processing enzymes, endogenous or secreted by pathogens are also included in the definition of peripheral proteins.

Unlike protein-protein or protein-ligand interactions, interfacial binding sites of peripheral proteins are poorly characterized in terms of amino acid composition and structural patterns. Embedded and transmembrane proteins contain well defined regions of hydrophobic surface, clearly identifying their membrane interacting segments. This is seldom the case for peripheral membrane proteins. Currently the prototypical peripheral membrane binding site is described as displaying a combination of basic and hydrophobic amino acids [7, 8]. Attempts to characterize the energetics of membrane binding has mostly focused on electrostatic complementarity of peripheral proteins with the charged surfaces of membrane [9], rather than on the desolvation of hydrophobes which is more difficult to isolate in theoretical treatments. Nevertheless the predictive power of implicit membrane models in the prediction of membrane binding sites is a strong indication of the importance of the hydrophobic effect [10] in peripheral membrane binding. For example, Lomize *et al.* could correctly identify the experimentally known IBS of 53 peripheral peptides and proteins using a model that includes only hydrophobic, desolvation and ionization energy terms [11]. Yet in order to assert the generality of a protein-membrane binding mechanism, it is not enough to demonstrate its validity for a selected set of true positives, but it is also important to evaluate it on a control dataset.

**Fig 1:**
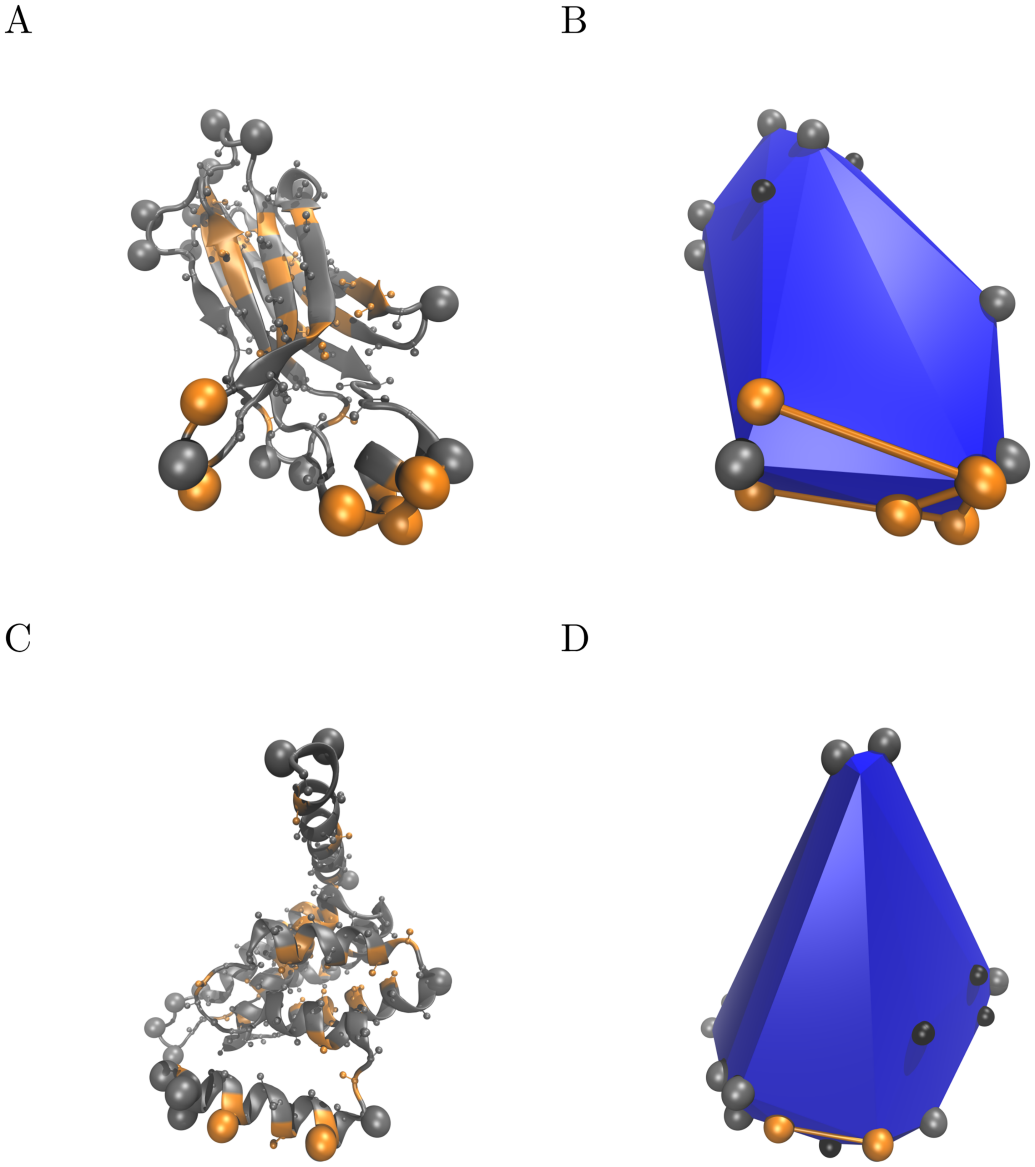
The definitions of ‘protrusions’ and ‘co-insertable protruding hydrophobes’. Panel A shows a cartoon representation of the C2 domain of human phospholipase A_2_ (PDB ID: 1RLW), and panel B shows the convex hull for the same protein. Panel C shows the structure of the ENTH-domain (PDB ID: 1H0A), which contains an amphipatic helix. The corresponding convex hull is shown on panel D. All C_α_- and C_β_-atoms are shown as spheres. Hydrophobes are coloured orange. The convex hull for the C_α_- and C_β_-atomic coordinates is shown in blue. All spheres visible on the convex hull representation are vertex residues. ‘Protrusions’ are defined as vertex residues with low local protein density, and shown as large grey spheres. ‘Co-insertable protruding hydrophobes’ are protruding hydrophobes that are adjacent vertices of the convex hull and are shown connected by orange lines. Small black spheres are at vertex residues that have high local density, and do therefore not meet the criteria for protrusions.

As both small hydrophobic patches and charged residues are frequently present on protein surfaces it is challenging to distinguish membrane binding sites from the rest of the peripheral membrane proteins surface solely relying on amino acid composition. There are indications that structural considerations may allow signatures of membrane interacting hydrophobes to be defined. Terms like *hydrophobic spikes* [12, 13] and *protruding loops* [11] have been used to describe membrane binding sites, prompting the idea of hydrophobes protruding from the protein globule. A close look at amphipathic helices, also motivates the concept of protruding hydrophobes. Amphipathic helices are characteristic of membrane-binding peptides and proteins. When such membrane binding helices exist, they are often found lining a protein, forming a cylindrical protrusion from the globule (e.g. ENTH domain of Epsin, PDBID: 1H0A [14], shown in Fig 1C and 1D). Yet, no generalization of protruding membrane binding sites has been proposed for peripheral membrane proteins.

The purpose of this contribution is to identify structural characteristics that distinguish exposed hydrophobes at membrane binding sites from those that are frequently found on any protein surface. We propose a simple definition that formalizes the concept of protruding hydrophobes, and which can be easily computed from the protein structure. This definition allows us to systematically investigate to what extent protruding hydrophobes are found on both binding and non-membrane-binding surfaces, and to identify structural criteria for recognizing exposed hydrophobes that are likely to be important for membrane binding.

A major obstacle in developing general association models for peripheral membrane proteins is the scarcity of experimentally verified binding sites, and detailed descriptions of binding orientations. Computational studies on the role of hydrophobes on membrane binding sites have been based so far on relatively small sets of proteins with known binding sites [10, 11, 15]. To get around this problem and to leverage the large number of proteins for which membrane binding has been identified without a detailed characterisation of the IBS, we perform a comparative statistical analysis of protein surfaces. Given classifications of proteins that identifies membrane binders, we compare peripheral membrane proteins with protein surfaces that are not membrane-binding and with more general reference proteins. With this we can extend our analysis to hundreds of protein families rather than the few dozens for which binding sites have been partially identified by experiments.

With our simple definition of structural protrusions, we perform a statistical analysis of protruding hydrophobes in a large protein structure dataset and our results support their general role in membrane association. We find that protruding hydrophobes can be used to strongly discriminate protein surfaces involved in membrane binding from those that are not. Hydrophobes are much more frequent on protruding sites of peripheral membrane proteins than in the reference dataset, and they have a strong tendency to cluster on positions that can simultaneously interact with the membrane.

## Results and discussion

Our formalisation of the concept of protruding amino acids is illustrated in Fig 1 and described in details in ‘Materials and methods’. In short, it relies on firstly identifying the convex hull (in blue in Fig 1) of a coarse-grained protein model consisting of only its C_α_- and C_β_-atoms. We then identify amino acids located at vertices of the convex hull which intuitively are good candidates to be inserted into a membrane without inserting other residues, and without deforming the protein backbone. The model thus implicitly assumes that (1) proteins interact with the membrane without appreciable conformational change, or prior to such change and (2) that the membrane is locally flat, which is a valid approximation in most cases [16]. In order to single out the amino acids that are most exposed to solvent, we identify amino acids (vertices) in regions of low protein density, defined as having a low number of neighboring atoms. Solvent accessibility is a necessary condition for the hydrophobic effect to contribute to binding. In addition, regions of low local protein density are also likely to cause less disruption of lipid packing upon membrane insertion. The model was formulated based on inspection of eight proteins for which ample experimental data is available. They are listed in the Supporting Information (Table B in S1 Text).

In what follows, we present results of the application of this model to characterise hydrophobic properties of protrusions in peripheral membrane proteins. We do this by comparing peripheral membrane proteins to a reference set of non-binding protein surface segments, and a reference set of typical protein surfaces. The reference set of non-binding surface segments (‘Non-binding surfaces’) is constructed from the solvent exposed regions of trans-membrane proteins and is intended to represent structures that do not interact with membranes. The reference set of typical proteins (‘Reference Proteins’) is constructed from a protein structural classification from which we have excluded proteins that are classified as membrane-interacting. This set is intended to represent more general representative protein surfaces, and includes an unknown frequency of peripheral membrane binders. Because our two reference datasets are obtained from different sources we cannot use exactly the same sets of peripheral proteins to compare them to. Specifically, we build two variants of the set of peripheral membrane proteins (‘Peripheral’ and ‘Peripheral-P’). These data sets are described in detail in ‘Materials and methods’. The main difference between those two sets is the modeling of quaternary structure which needs to be consistent with each of the reference datasets.

### Protruding hydrophobes in a dataset of peripheral membrane proteins

**Fig 2:**
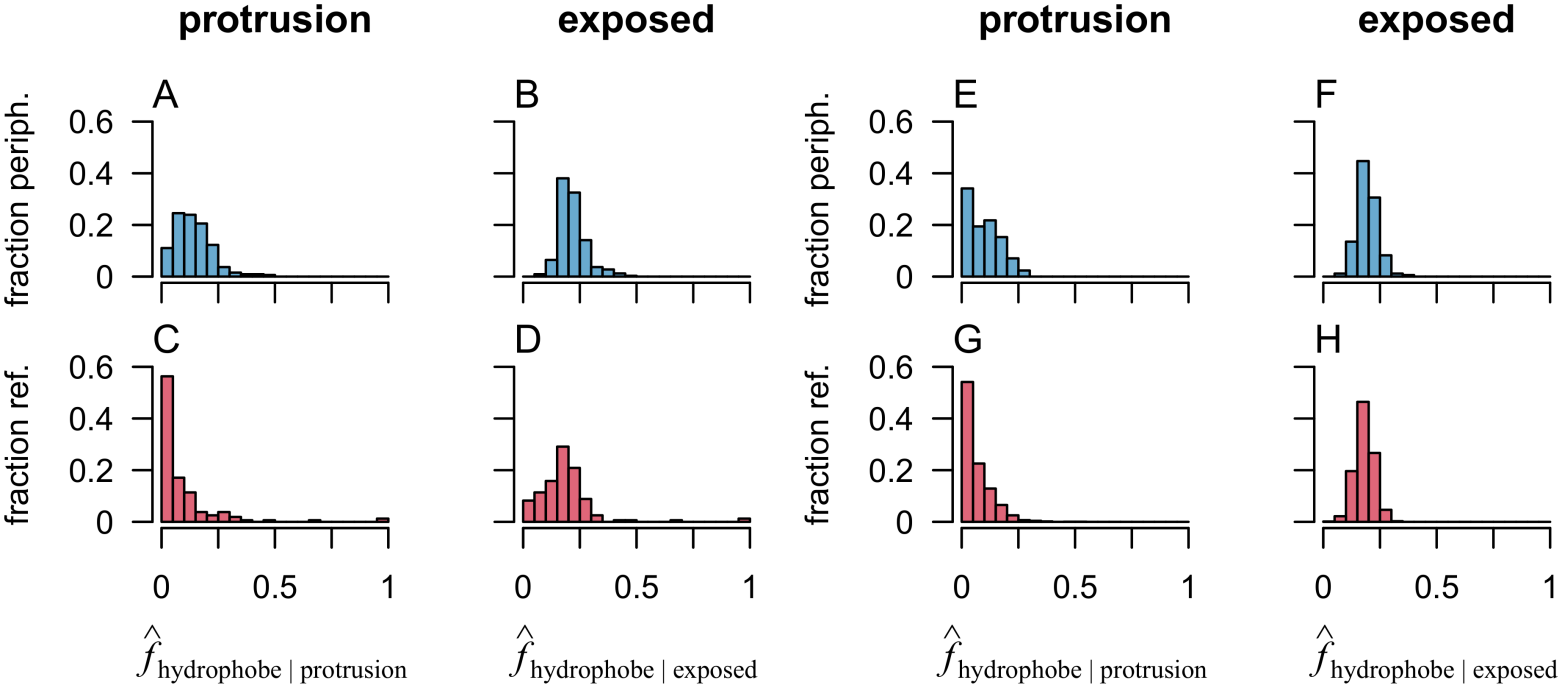
Hydrophobes are more common on protruding positions in peripheral proteins than in the reference sets. The plots show frequencies of hydrophobes on surface amino acids, both on protrusions (A, C, E, G) and among all solvent exposed amino acids (B, D, F, H) for peripheral proteins (blue) and the reference datasets (red). The horizontal axes show the mean fraction (Eq 1) of protrusions or solvent exposed amino-acids that are hydrophobic. The vertical axis shows the fraction of protein families for each set. Plots A-D show the comparison between the data sets ‘Peripheral’ and ‘Non-binding surfaces’, and E-H the comparison between ‘Peripheral-P’ and ‘Reference Proteins’.

First we calculated the frequency of hydrophobes on protrusions in peripheral protein families and compared it to the reference datasets. In Fig 2, we observe a stark contrast between the set of peripheral proteins and the non-binding surfaces (compare Fig 2A and 2C). Hydrophobes occur with high frequency and in almost all families on protrusions of peripheral proteins. In the reference set on the other hand, hydrophobes on protrusions are much less tolerated, reflected by a histogram mode of zero. While less pronounced, the distinction is also clear for the comparison with reference proteins (compare Fig 2E and 2G). Qualitatively, the frequency of hydrophobes on protrusions is similar in the two reference sets (Fig 2C and 2G) but the sets of peripheral proteins differ somewhat suggesting some sensitivity to quaternary structure modeling. For both comparisons however, this trend is specific for protruding positions and does not reflect a general difference in composition of surface exposed amino-acids between the data sets as shown by plots in Fig 2B, 2D, 2F and 2H. Indeed, if we consider the frequency of hydrophobes on all solvent exposed residues, the distributions look quite similar with both sets having histogram modes close to 0.2. This value is in agreement with the fraction of the surface of globular proteins typically reported to be hydrophobic (for instance 0.19 in Ref. [17]). The ‘Non-binding surfaces’ are in some cases very small, due to the way we ensure that these surfaces are not interacting with the membrane (see ‘Materials and methods’). While these small surfaces are relevant samples for calculating average frequencies, the fraction of hydrophobes on such surfaces can take more extreme values (close to zero or 1). For this reason the tails of the histograms for this reference set are somewhat fatter than those for the peripheral membrane proteins.

**Fig 3:**
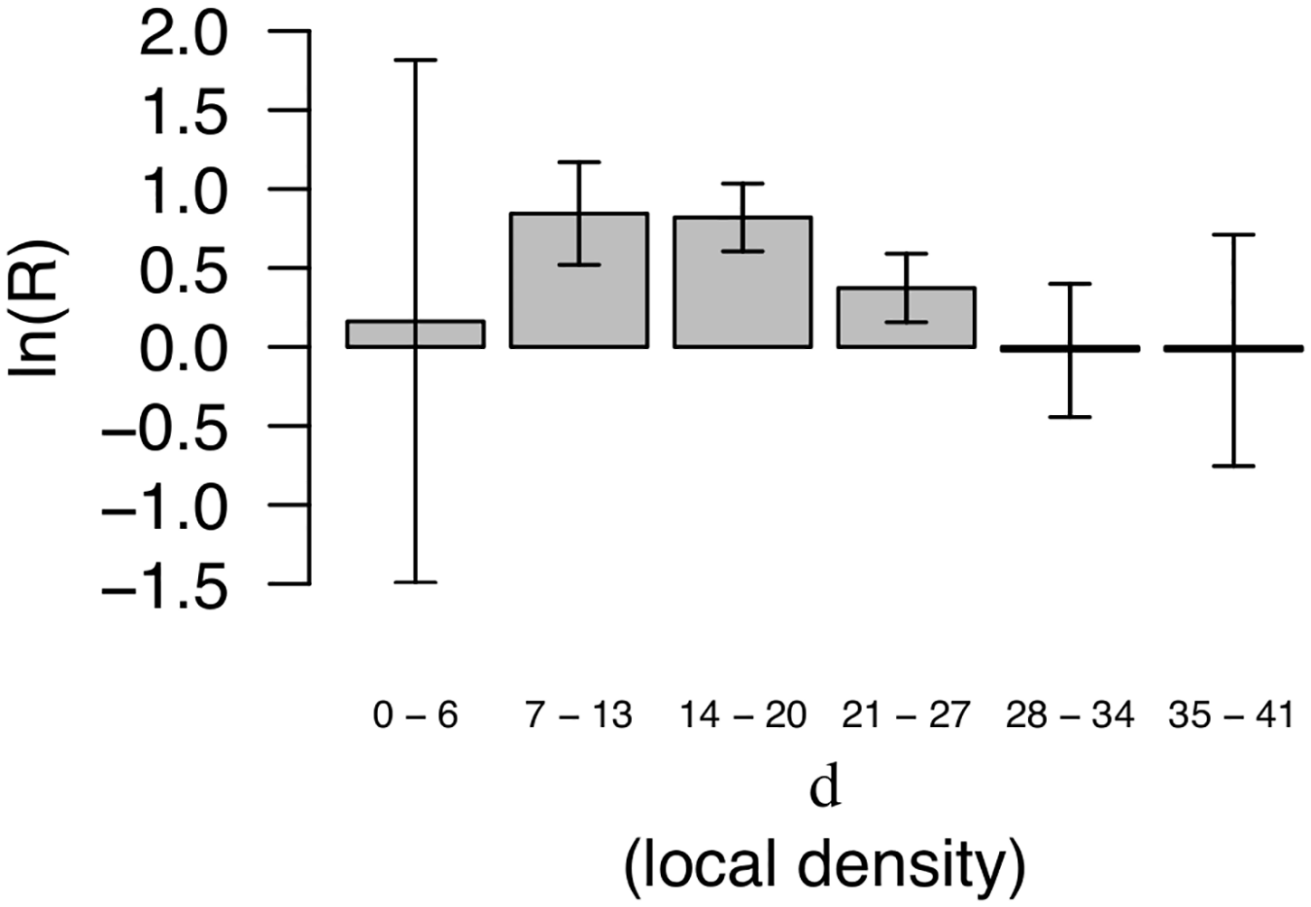
On peripheral proteins (‘Peripheral’ dataset) protrusions in low density regions are more often hydrophobes compared to the ‘Non-binding surfaces’. The plot shows the logarithm of the odds-ratio (Eq 10) comparing the frequency of hydrophobes on ‘vertex residues’ in peripheral proteins and non-binding surfaces. Positive values reflect higher frequencies in the peripheral proteins. The horizontal axis shows the protein density *d* around the protrusion, measured as the number of C_α_ and C_β_ atoms within 1*nm*. Vertex residues are all on the convex hull, but only the vertex residues with *d* < 22 are protrusions. The leftmost bar with *d* < 7 corresponds mostly to chain terminals. More precisely, the vertical axis shows $R(A,B,{\hat{F}}_{\text{hydrophobe}|\text{vertex}\cap l<d\leq u})$ where *A* denotes the dataset ‘Peripheral’, *B* the ‘Non-binding surfaces’, *l* and *u* denote the lower and upper limits of the ranges given on the vertical axis, and *d* is the local protein density defined in ‘Materials and methods’. Error bars are 95% confidence intervals.

Given the nature of our model the differences presented in Fig 2 are naturally ascribed to two factors; the accessibility of amino acids compared to other regions of the protein (they are vertices of the convex hull) and their low local protein density *d* defined as the number of neighboring C_α_- or C_β_-atoms (Cf. definition in ‘Materials and methods’). We here explore the dependence of this difference on *d*. In Fig 3 we show the difference between frequencies of hydrophobes in peripherals and the non-binding surfaces for different ranges of the local protein density *d*. The leftmost bar (0 ≤ *d* ≤ 6) corresponds to chain terminals. The other bars corresponding to ranges covered by our definition of protruding residues (7 ≤ *d* < 22) show that hydrophobic residues are more frequently found at vertex residues with low local protein density in the peripheral proteins. This also serves as an *a posteriori* justification for constricting our definition of protrusions to amino-acids with *d* < 22.

**Fig 4:**
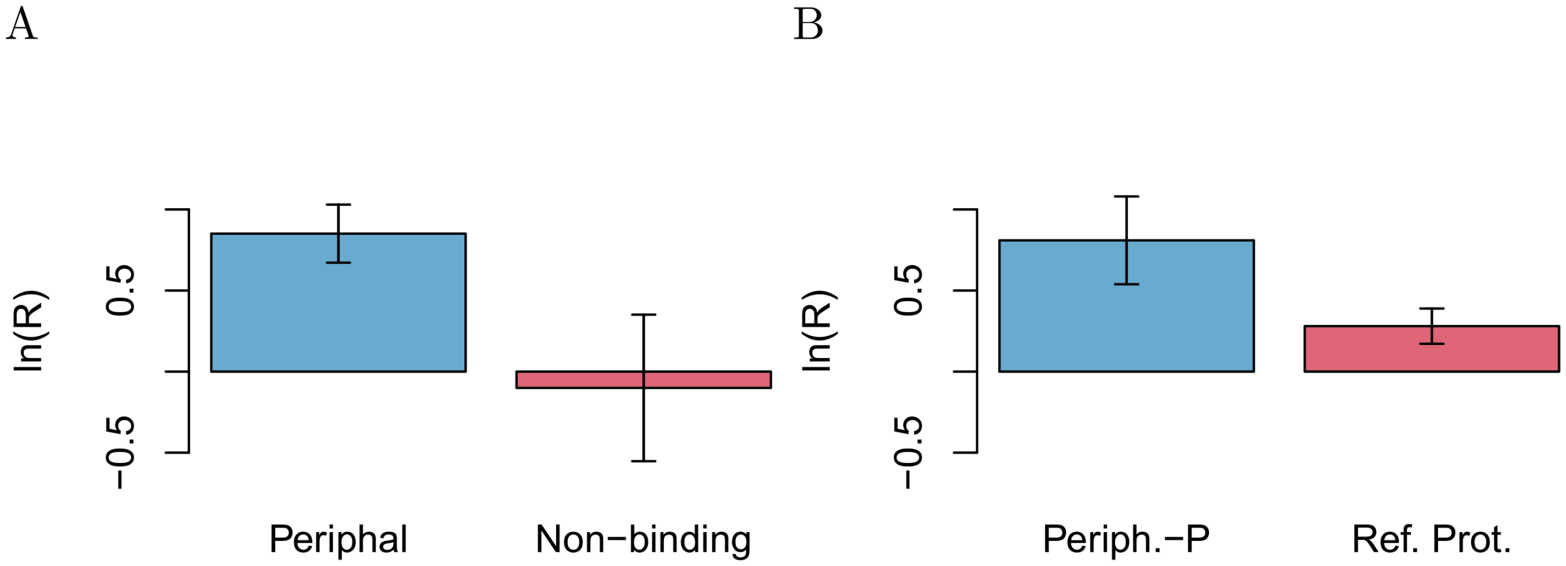
The ‘protruding hydrophobes’ tend to be ‘co-insertable’ in peripheral proteins. Panel A shows the comparison between the data sets ‘Peripheral’ and ‘Non-binding surfaces’, and B the comparison between the ‘Peripheral-P’ and ‘Reference Proteins’. The tendency for protrusions to be co-insertable is quantified by the weighted frequency of co-insertion (Eq 9), and is compared between each data set and a null model using the odds ratio (Eq 10). Positive values reflect higher frequencies of co-insertion than in the null model. More precisely we plot $R(set,null,{\hat{F}}_{\text{one},\text{both}}^{\text{pair}})$ where *set* represents the set of peripheral proteins (blue) and the corresponding reference set (red), and *null* represent their respective null models where hydrophobes have been relocated randomly among protrusions as described in ‘Materials and methods’. Error bars are 95% confidence intervals.

Assuming that the over-representation of hydrophobes on protrusions in peripheral membrane proteins stems from actual membrane binding sites, we expect those proteins to have more than one hydrophobic protrusion. We estimated the tendency of hydrophobic protrusions to be ‘co-insertable’ by calculating the weighted frequency of co-insertion (Eq 9) (Cf ‘Materials and methods’) for all datasets (Fig 4). We note that peripheral membrane proteins do indeed tend to have hydrophobes on co-insertable protrusions to a significantly larger extent than what would be expected from randomly scattering hydrophobes among protruding positions. This tendency is much lower for the ‘Non-binding surfaces’ even when considering the extremities of the error bars, which are wide precisely because there are very few protruding hydrophobes in this set. In the ‘Reference Proteins’ the analysis indicates that co-insertability is more common than in the null model, but far less so than in the Peripheral proteins.

**Fig 5:**
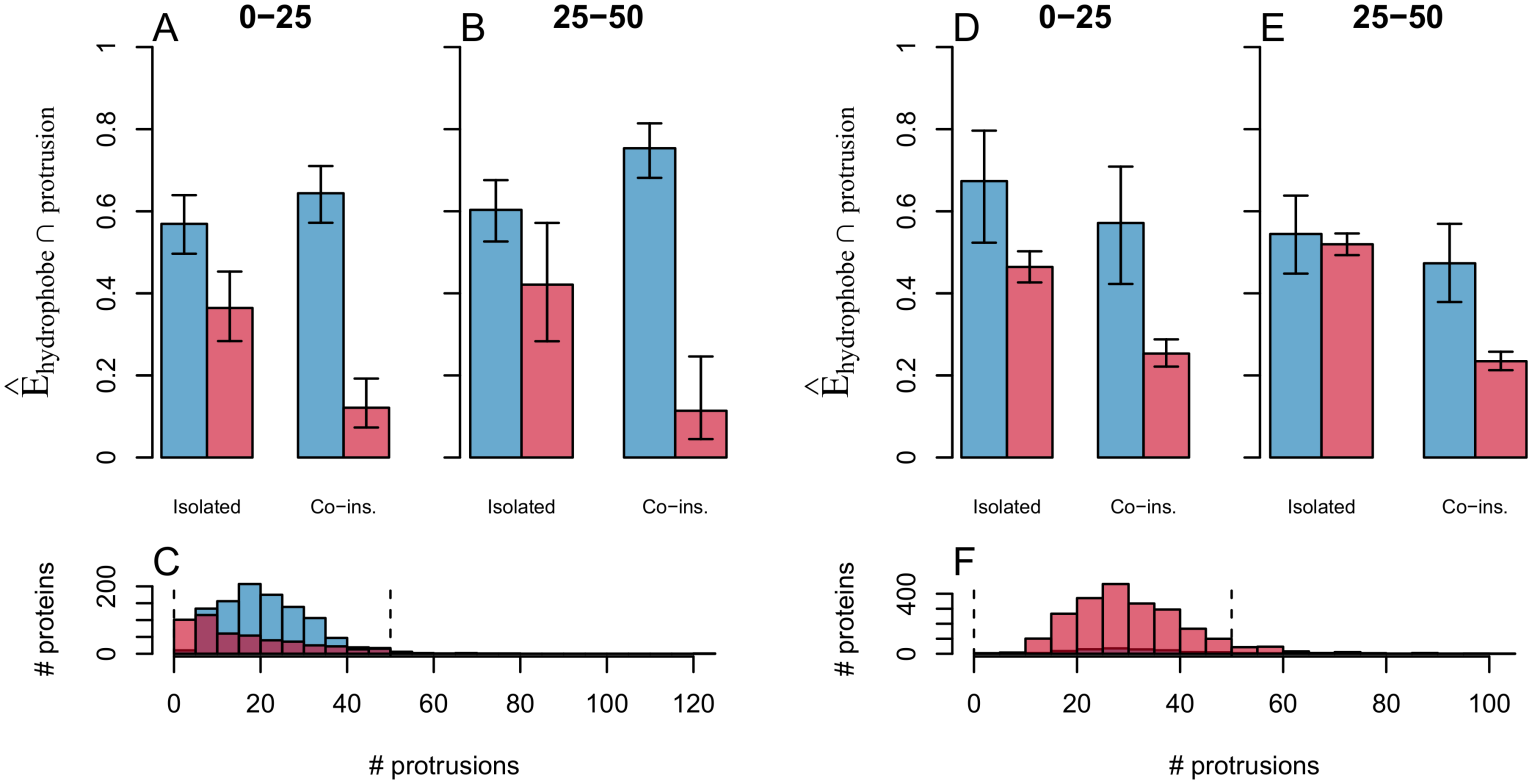
’Co-insertable protruding hydrophobes’ are common in peripheral proteins and rare in the reference sets. The plots show the occurrence of ‘co-insertable protruding hydrophobes’ on protein surfaces. Panels A-C show the comparison between the sets ‘Peripheral’ and ‘Non-binding surfaces’ and panels D-F the comparison between ‘Peripheral-P’ and ‘Reference Proteins’. Panels A, B, D, and E show the weighted fraction (Eq 5) of proteins that have protruding hydrophobes in the peripheral proteins (blue) and the reference sets (red). We differentiate here between protrusions that have at least one co-insertable protruding hydrophobe (labeled “Co-ins.”), and those that have not (labeled “isolated”). The analysis is done separately for two groups of proteins according to the total number of protrusions on the protein surface ([0, 25〉 in panels A and D, [25, 50〉 in panels B and E). Panels C and F show the frequency distribution of the total number of protruding residues (“# protrusions”) for all proteins. The selections analysed in panels A, B, D, and E are found between the dashed lines in panels C and F. Error bars in panels A, B, D, and E are 95% confidence intervals.

We further explore the degree of co-insertability of the hydrophobic protrusions present in our datasets. We seek to evaluate to what extent co-insertable hydrophobic protrusions can be used to discriminate likely peripheral membrane binders from other proteins. Fig 5 shows the fraction of proteins in each dataset that have at least one pair of co-insertable hydrophobic protrusions (labelled ‘Co-ins.’) and the fraction of proteins that have at least one isolated hydrophobic protrusion (i.e. a protrusion that does not satisfy the criteria that define ‘co-insertability’). While we do see some discrimination between the data sets in the case of isolated protruding hydrophobes, the co-insertable ones prove to be very strong indicators of which proteins surfaces are membrane binding. As the coincidental occurrence of such properties increase with the size of the protein surface, we have grouped the proteins by total number of surface protrusions (regardless of hydropathic properties). We do however see no appreciable difference between the proteins of size 0–25 and those of size 25–50. We consider the fraction in the reference sets to be a reasonable estimate of a false positive rate for predicting membrane binding function based on the presence of co-instertable protruding hydrophobes. The reference proteins (Fig 5D–5F), indicate a false positive rate in the range of 20%–30%. The lack of membrane interaction is not asserted for this set, and we do expect it to contain some proteins with undetected or unclassified membrane binding. The false positive rate is around 12% for the non-binding surfaces (Fig 5A–5C) but with a smaller sample size this estimate comes with somewhat higher error bars. Around 64% and 75% of the peripheral membrane proteins in the respective size-groups have co-insertable protruding hydrophobes. In line with the previous analyses (Figs 2 and 4) the predictive power is somewhat weaker for the ‘Peripheral-P’ dataset compared to ‘Peripheral’. We interpret this as a dependence on quaternary-structure modeling, which is corroborated by a dedicated analysis presented in the Material and methods section (Fig 11). We consider the manually curated oligomeric states to be more reliable and therefore expect the peripheral proteins presented in Fig 5A–5C (*Peripheral* dataset) to better represent actual proteins. In order to evaluate how common co-insertable protruding hydrophobes are as membrane-interacting motifs we will assume the rate of occurrence in the set ‘Peripheral’, and conservatively assume a frequency of occurrence on non-membrane interacting sites around 20%. This is consistent with both extremes of the 95%-confidence intervals in the non-binding surfaces (Fig 5A–5C) and the estimate from the reference proteins (Fig 5D–5F). Even when considering that as much as 20% of co-insertable protruding hydrophobes might not be membrane interacting we still expect a rough estimate of around half of the analysed membrane binders to have this motif at their membrane-interacting sites.

### Protruding hydrophobes vs. experimentally verified membrane-binding sites

**Fig 6:**
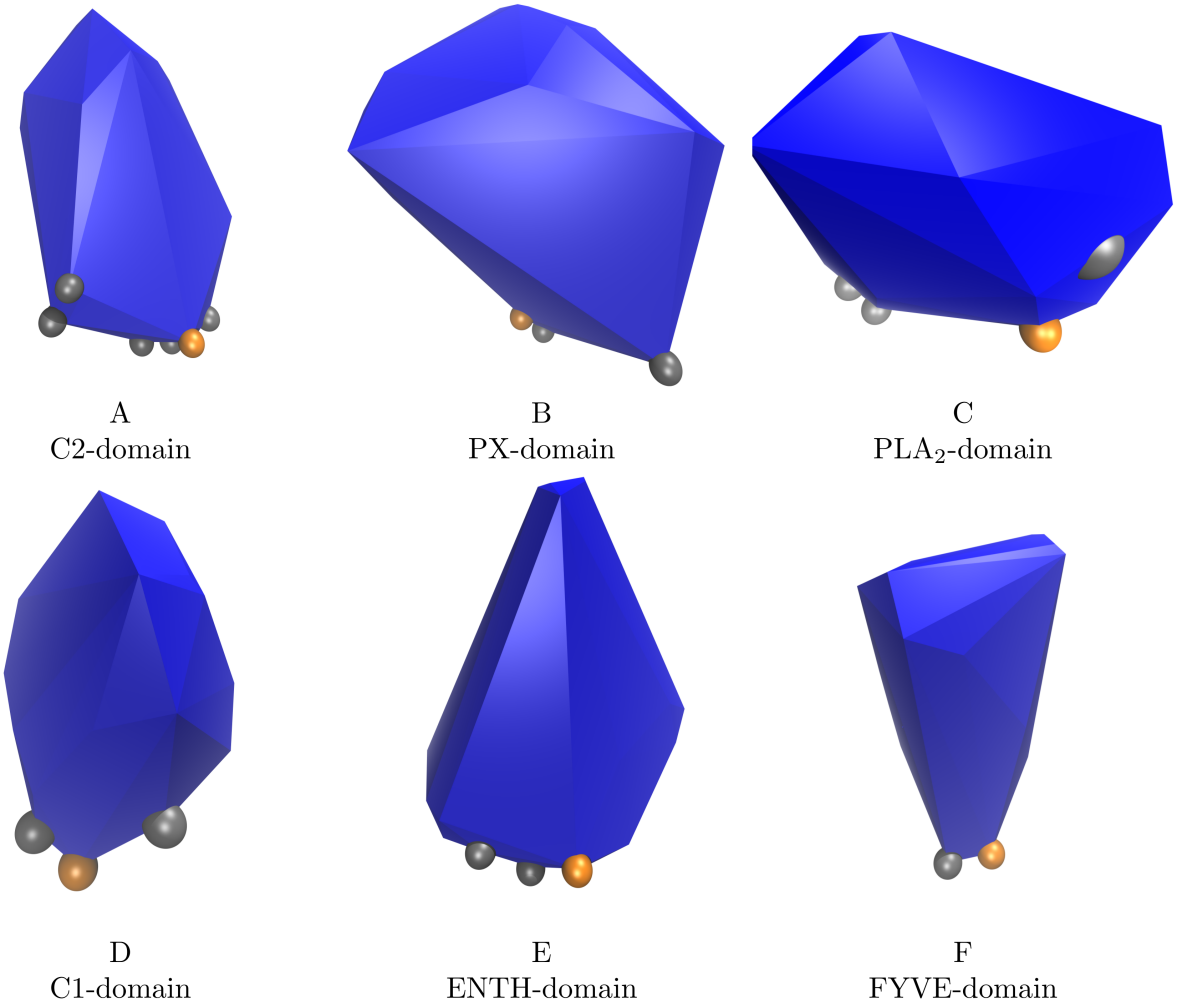
Protruding hydrophobes are found on the membrane binding sites of well known membrane binding domains. The figure shows the convex hull (in blue) of the C_α_ and C_β_-atoms of selected peripheral membrane binding domains. The C_β_-atoms of ‘the Likely Inserted Hydrophobe’ are shown as orange spheres and C_β_-atoms of experimentally identified membrane-binding residues as gray spheres. The Likely Inserted Hydrophobe is an amino acid that has been experimentally verified to be a membrane binding residue for A, B, D and F. For C and E the Likely Inserted Hydrophobe is located in the same area as the residues identified by experiments. **A**: C2 domain of human phospholipase A2 (PDBID: 1RLW [18]); **B**: PX domain of P40PHOX (PDBID: 1H6H [19]); **C**: snake phospholipase A2 (PDBID: 1POA [20]); **D**: C1 domain of protein kinase C δ (PDBID: 1PTR [21]); **E**: Epsin ENTH domain (PDBID: 1H0A [14]); **F**: FYVE domain of yeast vacuolar protein sorting-associated protein 27 (PDBID: 1VFY [22]).

**Fig 7:**
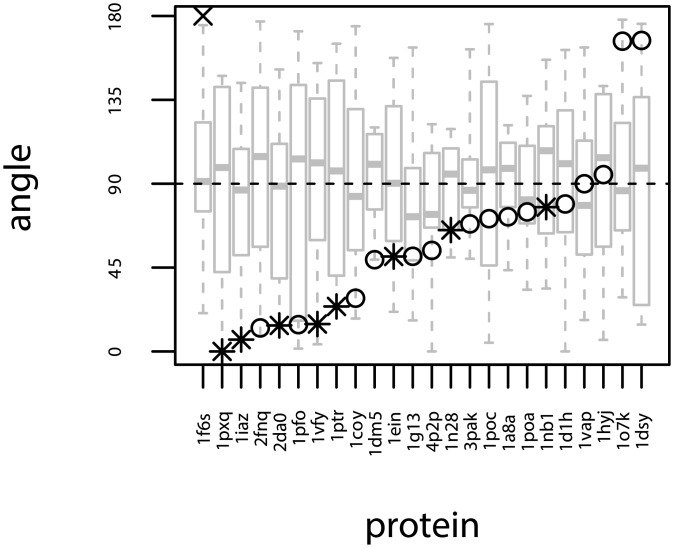
Protruding hydrophobes predict experimentally verified binding sites. The figure shows comparisons of predicted binding residues (‘the Likely Inserted Hydrophobe’) with experimentally verified binding sites for a manually curated dataset of 24 proteins (listed in S2 Table). The vertical axis corresponds to values of the angle (Eq 11) comparing the two vectors connecting the center of the protein with either the predicted or known binding sites. Smaller angles imply better agreement between prediction and experiment. Asterisks (*) mark proteins where the Likely Inserted Hydrophobe is an amino acid experimentally identified to be interacting with the membrane. The grey boxplots show the distribution of angles when the known binding site residues are compared to all protruding amino acids on the protein. 1iaz is analysed in its soluble monomeric state, while it forms a transmembrane pore upon oligomerisation. The structure of the Bovine *α*-lactalbumin (PDBID: 1F6S) has no identified protruding hydrophobes and is marked with a cross at 180°.

The analysis presented in Figs 3 and 5 suggests that the concepts of protruding hydrophobes and co-insertability can be used to identify membrane binding residues. Based on these results we seek to define a predictor of membrane binding sites. We define ‘the Likely Inserted Hydrophobe’ as the protruding hydrophobe with the highest number of co-insertable protruding hydrophobes and lowest local protein density, as defined in ‘Materials and methods’. Fig 6 illustrates that this simple definition is able to identify binding sites on modular membrane-binding domains: C1, C2, PX, ENTH, PLA2 and FYVE. For most of these cases, the Likely Inserted Hydrophobe has in fact been experimentally indicated to contribute to membrane binding. For the other examples, it is clearly positioned close to the experimentally identified binding site. A more quantitative comparison between predicted and verified membrane interacting residues is complicated by the sparsity of negative assertions from either methods. Experiments aiming at identifying membrane-binding sites will usually only target some of the amino acids suspected to belong to the membrane binding residues, and usually not conclude on other amino acids. To the extent non-binding amino-acids are investigated or revealed by the mutation of putative membrane binding residues, interpretation of results in this context is also less straightforward as the absence of interaction of an amino-acid with the membrane does not strictly preclude it from being located close to a binding site. Similarly the Likely Inserted Hydrophobe is by definition only one residue and provides no negative prediction of which amino acids do not bind the membrane. We can however make a rough, but well defined, comparison by computing the angle between the vectors connecting the protein center with respectively the mean position of the membrane interacting residues identified in experiments ($t_{I_{e}}$), and the Likely Inserted Hydrophobe ($t_{I_{p}}$, See Eq 11). While this comparison does not provide a quantitative evaluation of whether experimentally determined IBS and predicted residues match exactly, it allows us to separate proteins where the predicted and verified residues are “on the same side” of the protein ($∠t_{I_{e}}t_{I_{p}}<\;90{}^{\circ}$) from those where they are not. We show on Fig 7 such a comparison for proteins whose binding sites are experimentally determined. This is a coarse approximation to the protein orientation, which is sensitive to both protein shape, the selection of residues included in the partial biding sites, and any difference in backbone conformation between bound and unbound protein. Even so, we do expect that wrong binding site predictions should provide angles in the entire range from 0° to 180° with roughly uniform probability. But, we observe that almost all angles are sharper than 90°, indicating a reasonable agreement with experimental data. We also observe a similar range of angles for cases where the membrane interaction of the Likely Inserted Hydrophobe has been experimentally verified (marked with asterisks (*) in Fig 7) and the cases where it has not. We would like to emphasise at this point that the Likely Inserted Hydrophobes that are not yet found to be membrane interacting might very well never have been tested. We also calculated all angles between the set of experimentally identified residues and protruding amino acids of all kinds. These results are displayed as box-plots in Fig 7. While they vary a bit between families we note that all medians are close to 90°, confirming that the statistical expectation for protrusions in general is to have roughly equally many observations larger than and smaller than 90°. Interestingly, the Bovine *α*-lactalbumin, for which we find no protruding hydrophobes, is analysed in its crystallised form while it is known to bind membranes in a molten globule state [23].

We provide as Supporting Information the complete list of amino acids experimentally identified as being part of membrane binding sites (Table B in S1 Text). It overlaps with the list provided by Lomize *et al.* [11], but sometimes differ in exactly which amino acids are included, as we include indicated membrane interacting residues even when they are not inserted in the hydrophobic core of the membrane.

### Protruding hydrophobes on predicted membrane binding sites

**Fig 8:**
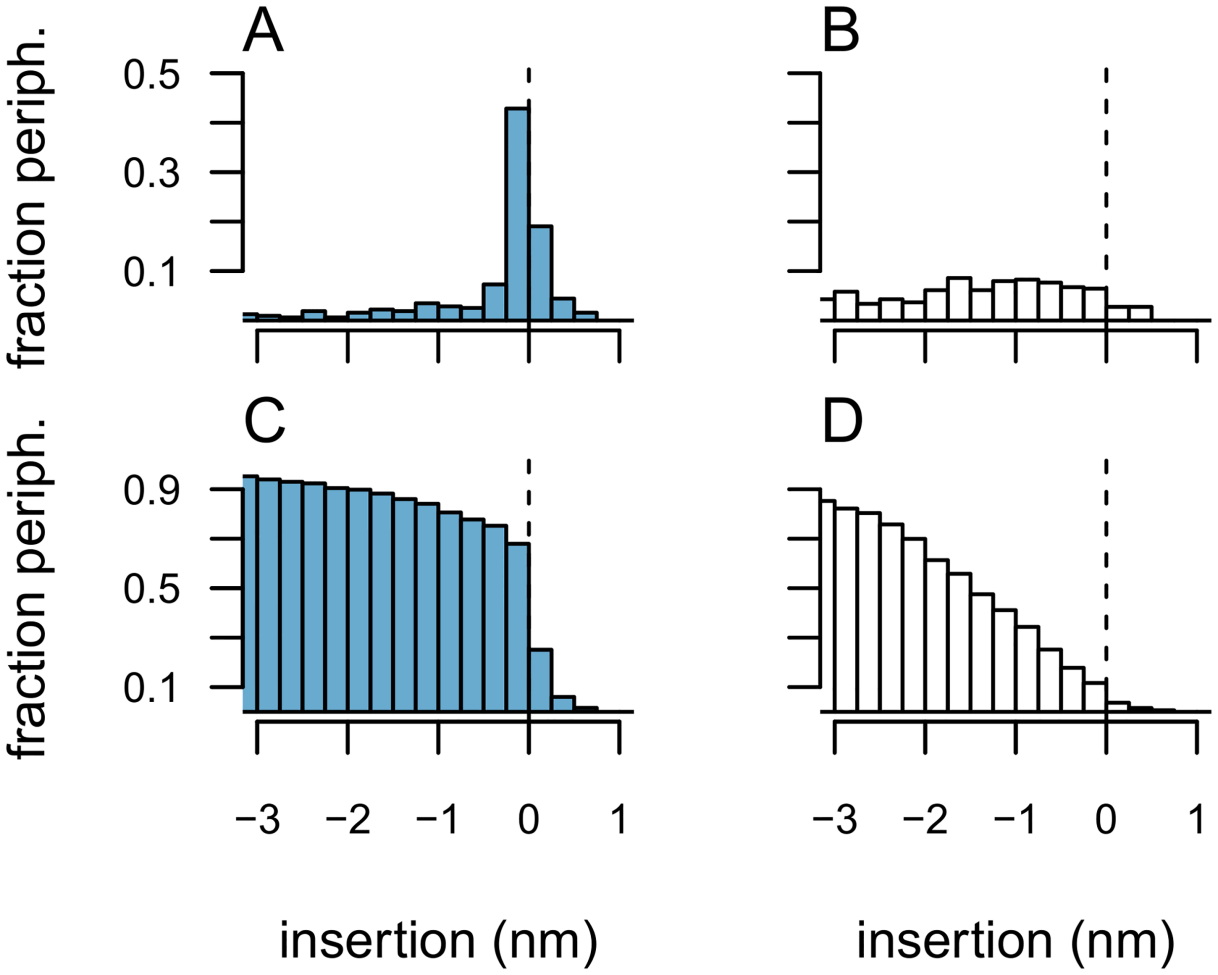
Comparing predictions based on protruding hydrophobes with the predicted IBS in the Orientation of Proteins in Membranes (OPM) database. The plots show the distributions of the median ‘insertion coordinate’ from OPM for ‘the Likely Inserted Hydrophobe’ in each family (measured at the C_α_-atom, ‘Peripheral’ dataset). Values greater than or equal to zero correspond to atoms positioned in the hydrophobic core or at the boundary. Hence insertion coordinate values close to zero indicate agreement with OPM. Panels A and C show data for the Likely Inserted Hydrophobes and panels B and D for a null model of randomly selected ‘protruding’ residues. Panels C and D show cumulative histograms (accumulated with decreasing insertion coordinate).

The continuum-model presented by Lomize *et al.* [24] forms the basis for a systematic effort to predict binding orientations for peripheral membrane proteins. The OPM database [25] provides prediction of spatial arrangements of membrane proteins with respect to the lipid bilayer for a selection of peripheral membrane proteins. We here investigate to what extent protruding hydrophobes are captured by the model proposed by Lomize *et al.* We identify The Likely Inserted Hydrophobe for each of the proteins in our dataset and extract the OPM predicted insertion coordinate of its C_α_-atom. The ‘insertion coordinate’ of an atom measures its depth of insertion into the hydrocarbon region of the membrane model and is thus positive for atoms located in the hydrocarbon core and negative for atoms located on either side of the membrane including the interfacial region (Cf. ‘Materials and methods’). Fig 8 shows histograms of the median insertion coordinate of the Likely Inserted Hydrophobes identified in each family. A clear majority of those residues are located close to the interface of the membrane model in the OPM-predictions (Fig 8A) and 75% of the families in the set of peripheral membrane proteins have the median insertion coordinate for the Likely Inserted Hydrophobe within a margin of 0.5 nm from the membrane. This fraction is similar to the estimated fraction of proteins that have co-insertable protruding hydrophobes (Fig 5A and 5B). We allow this margin of 0.5 nm to compensate for the assumptions of rigid protein, flat membrane, and the distance between C_α_-atoms and side-chain atoms. Fractions for other margins can be read from the cumulative histogram shown in Fig 8C. By representing position with the insertion coordinate we effectively project residue coordinates onto the membrane normal. We therefore do not expect surface amino acids to be uniformly distributed along the insertion coordinate axis and present control statistics for randomly chosen protruding amino acids of all hydropathic properties (Fig 8B and 8D). It appears clearly that the high number of Likely Inserted Hydrophobes close to the membrane model is not an effect of having more protein at that location.

### Structure and amino acid composition at hydrophobic protrusions

**Fig 9:**
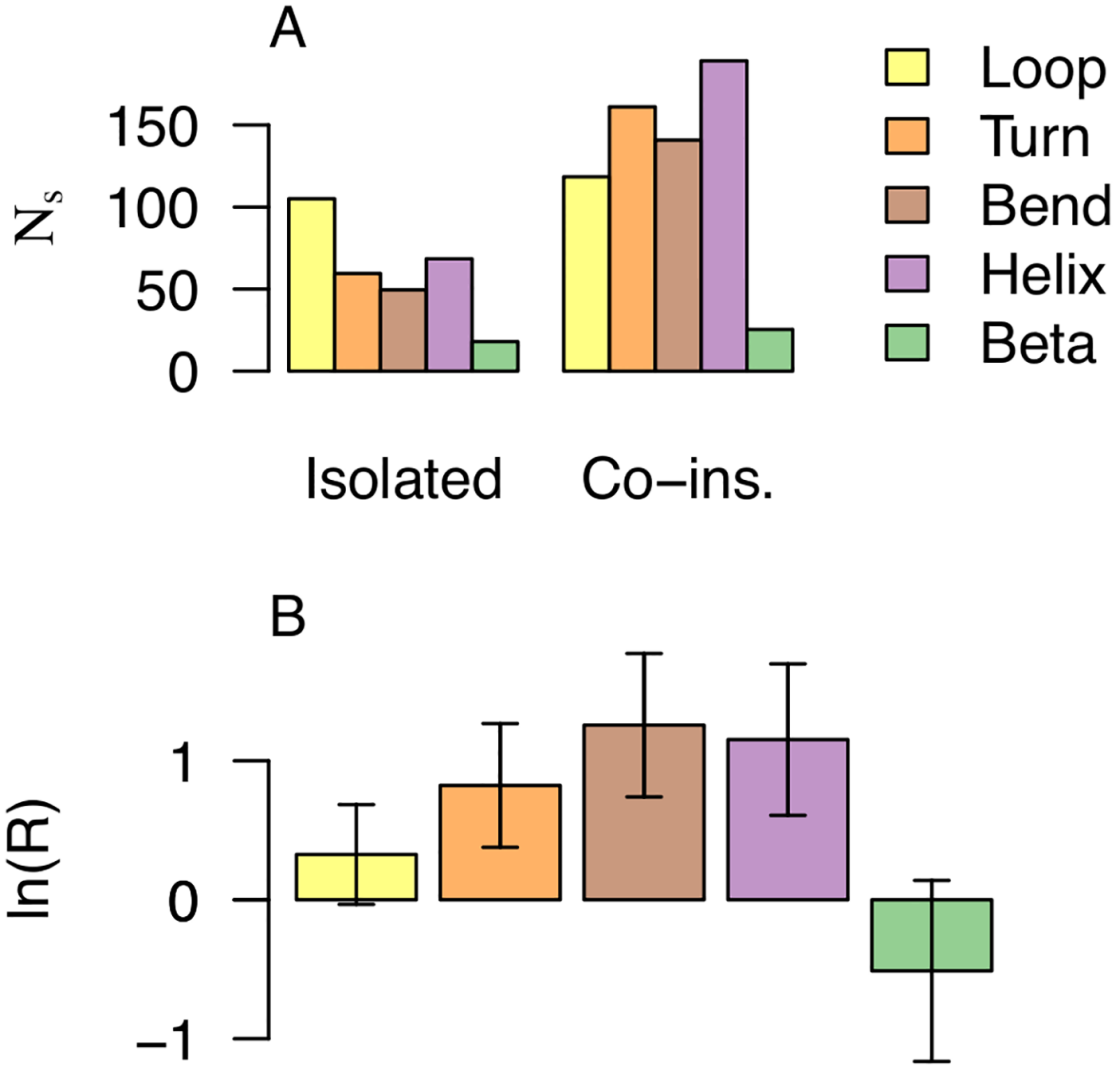
Hydrophobic protrusions in peripheral proteins are more frequent on turns, bends and *α*-helices, compared to the reference set (‘Non-binding surfaces’). Panel A shows the weighted number(Eq 2) of ‘protruding hydrophobes’ associated with the different types of secondary structure elements. We have differentiated between protrusions that have at least one co-insertable protruding hydrophobe (right, labeled “Co-ins.”), and those that have not (left, labeled “Isolated”). Panel B compares the weighted frequencies (Eq 4) of hydrophobes on protruding secondary structures between the peripheral membrane proteins and the reference set using the odds ratio (Eq 10). Positive values reflect higher frequencies in the peripheral proteins; panel A shows the values *N*_hydrophobe|protrusion∩*sse*_ and panel B the comparisons $R(A,B,{\hat{F}}_{\text{hydrophobe}|\text{protrusion}\cap sse})$ where *A* denotes the peripheral proteins, *B* the reference set and *sse* specifies the secondary structure (see color legend). Error bars in panel B are 95% confidence intervals.

The analysis presented in Fig 3 indicates that the ability to discriminate the data sets based on the frequency of hydrophobes on protrusions gets lower as the local protein density gets higher. Local protein density of a protrusion is dependent on secondary structure elements with loops, turns and bends being those that intuitively favor low local protein density. These secondary structures typically mark a clear change in direction of the backbone trace, where the neighbouring residues ‘make way’ for the protruding hydrophobe. Fig 9A shows which secondary structure elements the protruding hydrophobes are associated with in the set of peripheral proteins. We note that loops, turns and bends are indeed abundant but so are also helices and not beta-strands. Fig 9B shows a comparison with the reference data set (‘Non-binding surfaces’). We see that protruding hydrophobes on turns and bends are not only common in the peripheral membrane proteins as we saw in Fig 9A, but that they are also significantly more frequent than in the reference set. Interestingly, this is not the case for loops. Turns and bends are by definition structural elements with restricted flexibility [26] compared to loops, which are here defined as the absence of any of the other secondary structure definitions (equivalent to ‘coil’). We expect the latter category to contain less regular, more flexible structures. We speculate that turns and bends provide rigid scaffolds for exposing hydrophobic side chains, which might otherwise rearrange to desolvate when exposed to solvent. We also expect a similar property of rigid scaffolding from amphipathic helices, which is an established motif for membrane association. Fig 9 illustrates however that protrusions are not dominantly helices, confirming that the concept of protruding hydrophobes provides a useful generalisation for the shapes of membrane-binding sites.

**Fig 10:**
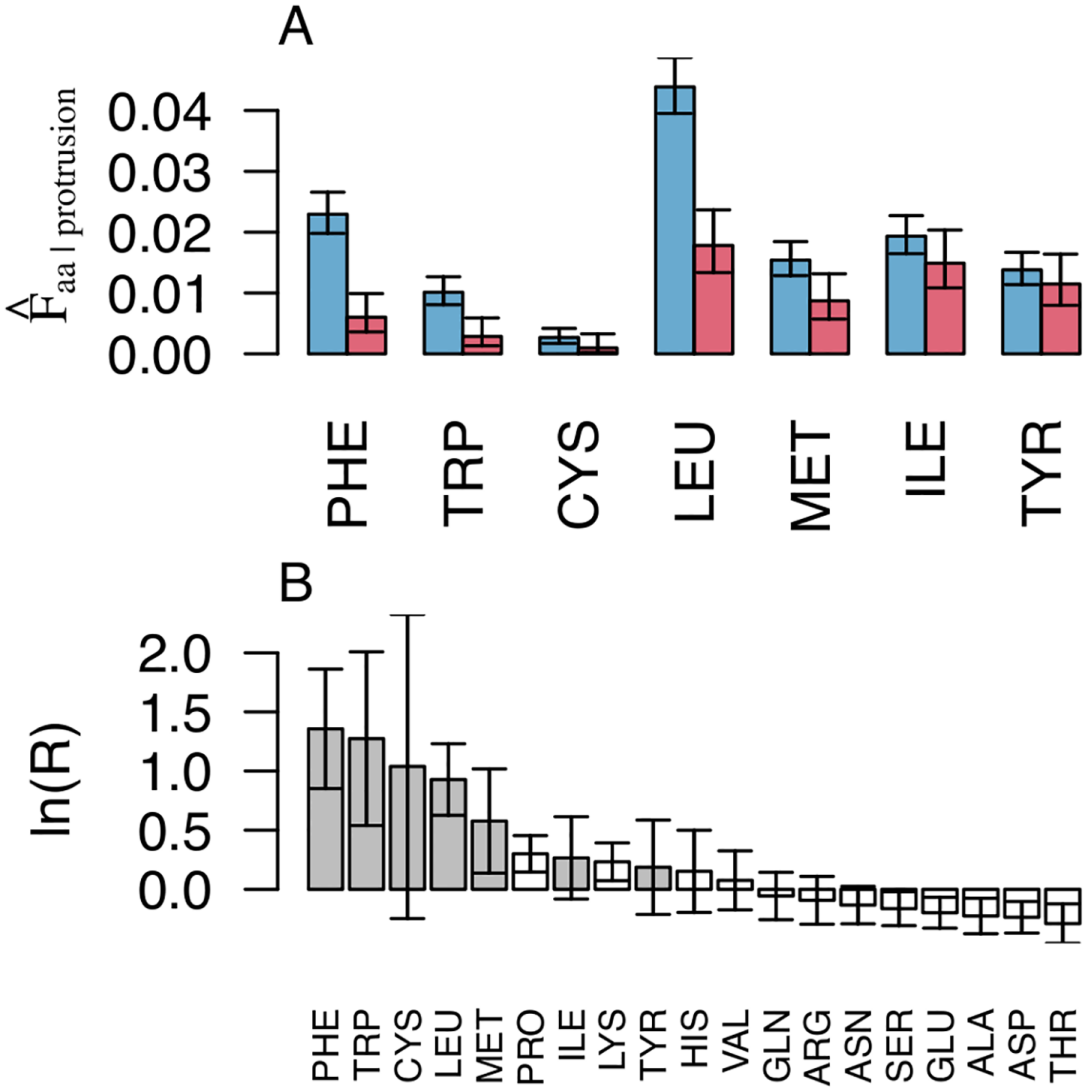
Large aliphatic and aromatic side chains are over-represented on protrusion on peripheral proteins. Panel A shows the weighted fractions (Eq 4) of hydrophobic amino acids on protrusions from peripheral proteins (blue) and from proteins in the reference set (red, ‘Non-binding surfaces’). In panel B, the contrast between the two sets is quantified by the odds ratio (Eq 10), so that positive values reflect higher frequencies in the set of peripheral proteins than in the reference set. More precisely the vertical axis denote $\text{ln}\;R(\text{peripheral},\text{reference},{\hat{F}}_{aa,\text{protrusion}})$ with *aa* representing each of the standard amino acids. Error bars are 95% confidence intervals.

For purposes of isolating the structural component of hydrophobic membrane association we have until now used a dichotomous definition of hydrophobicity based on signs of free energy of transfer determined by Wimley and White [27] (leucine, isoleucine, phenylalanine, tyrosine, tryptophan, cysteine and methionine have been considered to be hydrophobic). Yet, we do expect different amino acids to have varying contributions to the free energy of binding. We have therefore also assessed the relative importance of different amino acids for discriminating between our sets. Fig 10B shows the comparison of the frequencies of different hydrophobic amino acids on protrusions in the set ‘Peripheral’ and the set ‘Non-binding surfaces’. Analysis of the other two sets can be found as Supporting Information (S1 Text). As expected we find non-polar residues with large aliphatic or aromatic side chains to be much more frequent at the protrusions of peripheral proteins than on the non-binding surfaces. While the error bars in Fig 10B are not corrected for multiple testing, the signal for the hydrophobes as a group is quite clear. They all occur as over-represented in the set ‘Peripheral’ and the odds-ratio is much larger for phenylalanine, leucine and tryptophan than for any of the amino-acids that are over-represented in the set ‘Non-binding surfaces’. Analysis of the other two sets can be found as Supporting Information (S1 Text). Recall that ln *R* (Eq 10) is symmetric around 0, so the magnitude of the bar representing phenylalanine on one end, can be directly compared to that of the bar representing threonine in the negative direction. Tyrosine on the other hand discriminates the sets poorly compared to its high hydrophobicity score in the Wimley-White scale. We consider this a possible consequence of the orientational restrictions on the binding sites of peripheral membrane proteins. The typical orientations consistent with shallow binding has the residue anchored above the membrane. This probably allows less freedom for the polar hydroxyl group of tyrosine to orient towards regions of higher water density, than it has in the peptides used for the Wimley-White experiments or in transmembrane proteins. We also note with interest that proline is among the residues that are somewhat over-represented in the set of peripheral proteins. In general prolines are conformationally important protein components that restricts the backbone with respect to its immediate neighbours along the peptide chain. They are therefore likely to promote local rigidity. They also serve to induce sharp changes in the backbone direction. We speculate that this would facilitate solvent exposure of neighbouring side-chains as discussed above. Specifically they are in general frequently found on turns [28].

### Comments on the protein model

The convex hull representation presents a useful abstraction of proteins for investigating surface properties of approximately rigid protein conformers interacting shallowly with an approximately flat membrane. The model enables statistical analysis of protein structures, which is prohibited by high-resolution models where model parameters and quality controls typically have to be made subjectively for individual protein-membrane systems. We have employed this abstraction specifically to quantify and understand aspects of hydrophobes in peripheral membrane binding. In order to isolate components contributing to membrane binding we have purposefully avoided complicating the interpretation with other known important factors such as electrostatics, conformational flexibility and even relative hydrophobicity. For the purpose of understanding the balance and complementarity between different contributions to membrane-binding and making more generic models it will be necessary to take these other factors into account in ways that allows decomposition of their contribution. In the framework of a non-energetic structural analysis as the one we present in this manuscript, it is natural to do that in terms of comparing presence -or absence- and location of predicted binding sites between protein models. Particularly, models of electrostatic binding are well developed and readily applicable to surface representations of rigid protein conformers. While complex energetic models or machine learning approaches can be expected to yield high performance in predicting membrane-binding properties of proteins, the kind of model presented here provides a clear interpretation of the resulting prediction (membrane-binding or not) and mechanistic information. This connection to expert knowledge is invaluable for interpreting automated classifications where the models can not be reliably parameterised against negative data, that is definitely non-binding proteins. The combined use of various binding-site indicators based on different generic binding models such as hydrophobic and electrostatic models can provide a much improved performance in such prediction while maintaining interpretability. Such an approach would also be useful for inference or interpretation of protein specificity towards particular lipid compositions of the interacting membrane.

### Conclusion

Protein-membrane interactions are typically studied *in vitro* or *in silico* and inference to their biological context have to carry over from greatly simplified membrane models. To make sense of such experiments and simulations, it is essential to formulate general models that explain protein association in terms of factors that are present in both model systems and the relevant *in vivo* counterpart. In pursuit of such general models for membrane recognition, we have formulated the concepts of protruding hydrophobes and co-insertability. We have analysed more than 300 families of proteins that are classified as peripheral membrane binders and identified this model to be a good fit for at least half of them, after cautiously correcting for conservative false positive rates estimated from the reference sets (Fig 5). The generality of the model is corroborated by three important points. Hydrophobes are clearly over-represented on the protrusions of peripheral membrane proteins (compare Fig 2A and 2C, and see Fig 3), they tend to locate on co-insertable protrusions (see Figs 4 and 5), and protruding hydrophobes are generally positioned consistently with experimentally identified binding sites (Figs 6 and 7). Amphipathic helices are already well known membrane binding motifs which our definition of protrusion is well suited to capture, whenever these are stably folded and exposed. We do however find that the majority of identified protruding hydrophobes are not helices (Fig 9A) and that hydrophobes are also highly over-represented on protruding turns and bends (Fig 9B). We therefore propose the concept of protruding hydrophobes as a useful generalisation upon binding motifs that are identified in terms of secondary structure.

Investigation of the interfacial binding sites of numerous peripheral membrane proteins has revealed the presence of hydrophobic amino acids and of basic amino acids such as arginines and lysines. This reflects the two universal traits of biological membranes; their hydrophobic core and anionic surface. Yet the focus on the electrostatic component of the free energy of transfer from water to membrane—often referred to as being long-range—has overshadowed the importance of hydrophobic contribution which is sometimes referred to as being short-range. The focus on electrostatic interaction is at least in part to be attributed to the difficulties in evaluating the hydrophobic contribution as opposed to for example, the computational tractability of continuum electrostatic models. In principle the contribution of hydrophobes to membrane binding can only be determined with a rigorous treatment of the hydrophobic effect, which requires very accurate treatment of large systems involving both protein, membrane and solvent. The mere presence of hydrophobes on the protein surface is to a large extent tolerated by non-membrane-binding proteins as well. For both hydrophobes and basic amino acids, it is challenging to determine when their presence on protein surfaces are coincidental, and when they are important for membrane binding. Moreover, amino acids on membrane binding sites are not typically strongly conserved [29] so modeling their generic binding modes is important both for relating binding sites between homologs and for understanding how additional factors determine differences in membrane specificities. Fortunately, as evident from the results presented in this contribution, the role of hydrophobes can often be understood in much simpler terms than what is required for an exact estimate of the energetics of the hydrophobic effect and their importance for membrane-binding can be inferred from comparative statistical analyses. The subtle considerations of protein structure encoded in our definition of protrusions, strongly distinguishes the small hydrophobic patches on peripheral membrane proteins from those on other protein surfaces. This provides reliable evidence to assume their importance for binding.

## Materials and methods

### Data sets

We have compiled four data sets, two versions of a set of peripheral proteins, and two different reference sets:

- ‘Peripheral’: A set of peripheral membrane binders obtained from the OPM-database [25] using the OPM quaternary structure models.
- ‘Peripheral-P’: A subset of ‘Peripheral’ where no protein overlap in terms of their SCOPe-family classification [30] and with quaternary structure predicted by PISA [31].
- ‘Non-binding surfaces’: A set of protein surfaces obtained from the solvent exposed regions of transmembrane proteins.
- ‘Reference Proteins’: A non-redundant set of proteins from 5 SCOPe-classes obeying the following conditions: (1) none of these proteins have a domain represented in OPM and (2) none of the proteins in the dataset have domains belonging to the same SCOPe-family (the same restriction as for ‘Peripheral-P’).

In our analysis ‘Peripheral’ is always compared to ‘Non-binding surfaces’, and ‘Peripheral-P’ to ‘Reference Proteins’.

‘Peripheral’ are all the proteins in OPM classified as type ‘Monotopic/peripheral’. While the OPM has strict criteria for inclusion, membrane binding is not asserted by experiment in all cases and the set might contain false positives. This data set is provided as Supporting Information (S1 Dataset).

The set ‘Non-binding surfaces’ consists of fragments of transmembrane complexes. We obtained these protein fragments from all proteins classified as type ‘Transmembrane’ in OPM. The fragments analysed are composed of all amino acids whose C_α_-coordinates are at least 1.5 nm from the hydrocarbon region of the membrane model (parameter *Z*_*HDC*_ in the OPM model [32]). We rely here on membrane models positioned by the OPM, which we deem reliable for transmembrane proteins. While the entire protein complex was considered when calculating structural properties, only the fragments meeting this distance criteria were considered in the statistical analyses. When these proteins interact with secondary membranes or interact with membranes of extremely high curvature, it is not captured by the OPM model and the assumption that these surfaces are not interacting with membrane may be violated. We have assumed that such issues are exceptional. This data set is provided as Supporting Information (S2 Dataset).

We do consider the assumptions mentioned above to be conservative. Inclusion of non-membrane-binding proteins in our set of peripheral membrane proteins would likely weaken any general signal from membrane binding proteins and inclusion of secondary membrane interactions sites in the reference set would probably inflate the number of hydrophobes on protrusions in that set.

All protein structures in these two sets are obtained by X-ray crystallography and NMR spectroscopy and we have assumed that at least the backbone coordinates are representative of the solvated state of the proteins. As the source of structural information for this database is the Protein Data Bank (PDB) [33] the relevant oligomeric state is not always determined. The curators of the OPM-database have decided on oligomer models, upon which we have relied for the sets ‘Peripheral’ and ‘Non-binding surfaces’. These are taken from PDBe [34] and generated by PISA [31] or obtained from literature as described by Lomize *et al.* [25].

Even if the solvent exposed regions of the proteins in the set ‘Non-binding surfaces’ are extracted after relevant properties for potential membrane interaction was calculated, we cannot exclude totally that the surface constructed reflect artifacts of the extraction of fragments from complete protein models. In addition we expect our analysis to be sensitive to quaternary structure modeling as oligomeric protein-protein interfaces may also contain exposed hydrophobic patches [35, 36]. As a quality control we therefore also performed an analysis ourselves relying solely on computationally predicted quaternary structures and complete protein structures. This is achieved by the comparison of ‘Peripheral-P’ and ‘Reference Proteins’.

The set ‘Reference Proteins’ is constructed from SCOPe [30] and is a subset of all PDB IDs determined by X-ray crystallography, with at least a domain classified in SCOPe [30] in the classes ‘All alpha proteins’ (sunid: 46456), ‘All beta proteins’ (sunid: 48724), ‘Alpha and beta proteins (a+b)’ (sunid: 51349), ‘Alpha and beta proteins (a/b)’ (sunid: 53931) or ‘Multi domain proteins’ (sunid: 56572). The exclusion of structures not determined by X-ray crystallography ensures the consistency of quaternary structure predictions. All PDB IDs that have one or more domains classified in the same SCOPe-family as any domain in the OPM-database [25] were excluded from the set. This excludes not only the peripheral membrane binders, but also any transmembrane protein found in the reference set used for our primary analysis. In order to avoid redundancy, we iteratively removed proteins with domains that share SCOPe-family classification with any other domain in the set, until there were no such shared classifications left. This process ensures that there is at most one representative for each SCOPe family in the set. We generated quaternary structure models using PISA [31] for all members of this set. While this data set consists of more complete protein surfaces than the dataset of ‘Non-binding surfaces’, it is intended to be a reference for typical protein surfaces and we do expect it to be a mix of both membrane interacting and non-interacting proteins. This data set is provided as Supporting Information (S4 Dataset).

The set ‘Peripheral-P’ was derived from ‘Peripheral’ for comparability with ‘Reference Proteins’. All structures not determined by X-ray crystallography were excluded and proteins with domains that share SCOPe-family classification with any other domain in the set were iteratively removed to avoid redundancy. Quaternary structure models were predicted using PISA. This data set is provided as Supporting Information (S3 Dataset).

A few structures meeting the criteria above were not included in the analysis for technical reasons including issues with formats of PDB files. After exclusion of these cases the final ‘Peripheral’ dataset contains 1012 protein structures classified into 326 families. The final set of ‘Non-binding surfaces’ contains 495 protein structures classified into 158 families. The final set of ‘Peripheral-P’ binders contained 170 proteins (or families) and the set ‘Reference Proteins’ contained 2250 proteins (or 2250 families).

**Fig 11:**
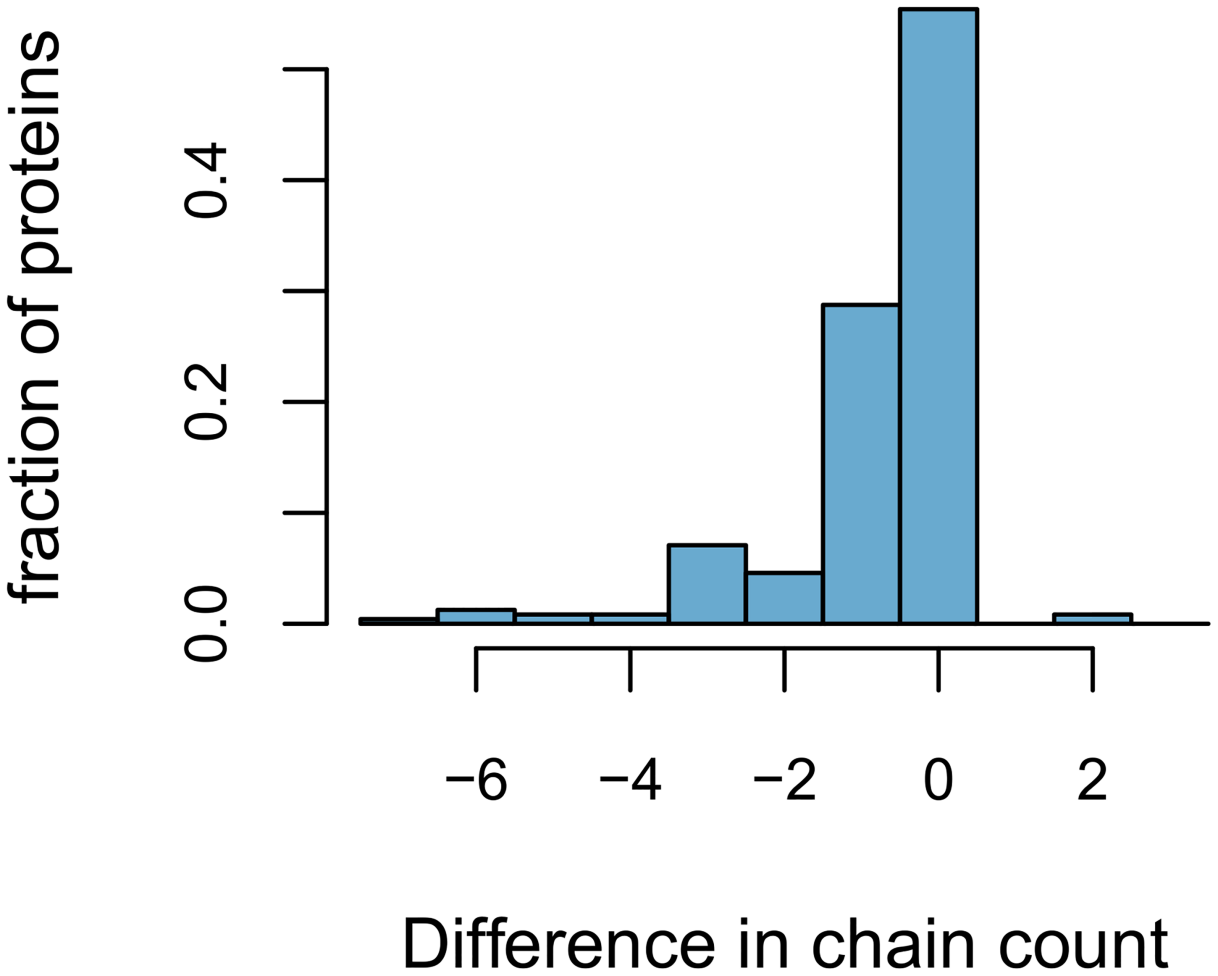
Differences in number of polypeptide chains between the protein models present in the dataset ‘Peripheral’ (quaternary structure model from OPM) and the models in ‘Peripheral-P’ (quaternary structure model predicted by PISA). The difference is calculated for each of the PDB IDs occurring in both datasets. When more chains are present in the PISA models, The difference (horizontal axis) is negative.

The two sets of peripheral proteins are both derived from OPM but ‘Peripheral-P’ is organized in a different classification than ‘Peripheral’ and retains fewer structures. In addition their quaternary structures, which are not completely determined by X-ray crystallography, are modeled differently. In Fig 11, we illustrate this difference in quarternary structure by showing the difference in the number of polypeptide chains present in the models belonging to each of the two sets.

Based on experiments reported in available literature [12, 23, 37, 38, 38–41, 41, 42, 42–70], we built a dataset of partially identified membrane binding sites on proteins with resolved structures. This set contains membrane interacting residues of 34 protein structures classified into 22 families. A detailed description is provided in the Supporting Information (Table B in S1 Text).

### Definitions

#### Structural characteristics of protein surfaces

We characterise the surface of proteins with different criteria designed to capture solvent ‘exposed’ residues, ‘protruding’ residues and ‘co-insertable’ protruding residues. The two latter are illustrated in Fig 1.

‘Exposed’ amino acids are defined as all amino acids that have a solvent accessible side-chain area greater than 0.2 nm^2^, as calculated with a probe with a radius of 0.14 nm, following the procedure described in Eisenhaber *et al.* [71] using van der Waals radi reported by Bondi [72].

We identify a ‘protrusion’ or a ‘protruding residue’ via the calculation of the convex hull of the C_α_- and C_β_-coordinates of the protein. The convex hull of a set of points *S* is the smallest possible convex set containing *S*. We define ‘vertex residues’ as residues whose C_β_-atom is a vertex of this convex hull. A ‘protrusion’ or a ‘protruding’ residue, is defined as a ‘vertex residue’ that also has low local protein density. For the purposes of this work, we will define the local protein density *d* of a residue, as the number of C_α_- or C_β_-atoms within a distance *c* of its C_β_-atom. We will designate a local protein density as low, if *d* < *n*, with *n* = 22 and *c* = 1 *nm*. These parameters were manually chosen based on a set of six different families of peripheral membrane proteins (C2-domain, PX-domain, Discodin domain, ENTH domain, Lipoxygenases and a Bacterial Phospholipase C). A list of these proteins are provided as Supporting Information (Table A in S1 Text).

We define two protrusions to be ‘co-insertable’ or a ‘co-insertable pair’, if the straight line connecting them is an edge of the convex hull polygon.

#### Hydrophobic residues

An amino acid is defined to be ‘hydrophobic’, or a ‘hydrophobe’, if it contributes favourably to membrane interface partitioning of peptides, as determined in the Wimley-White scale for interfacial insertion [27]. These amino acids are: leucine, isoleucine, phenylalanine, tyrosine, tryptophan, cysteine and methionine.

#### Secondary structure

We use DSSP definitions [73] for protein secondary structure. DSSP codes H, G or I are reported as ‘helix’, DSSP codes B or E as *β*, DSSP code T as ‘bend’ and DSSP code S as ‘turn’. All other residues are considered to be in ‘loops’.

#### Likely Inserted Hydrophobe

The ‘Likely Inserted Hydrophobe’ is defined as the protruding hydrophobe with the largest number of co-insertable protruding hydrophobes in a protein. Ties are resolved by choosing the likely inserted hydrophobe with the smallest local protein density *d*. Further ties are resolved by random selection, so that each protein has exactly one Likely Inserted Hydrophobe, unless it has no protruding hydrophobes at all.

#### Insertion coordinate

For comparisons with OPM predictions, we define the ‘insertion coordinate’ of atoms. This coordinate measures how deeply into the OPM membrane model an atom is inserted, and is therefore negative on the solvated side of the membrane. The membrane perimeter, where the insertion coordinate is 0, is the end of the hydrocarbon region. We identify this boundary as it is done in the model used to predict the OPM orientations, namely the planes where the volume fraction of total hydrocarbon is equal to 0.5. See Eq 2 in [32].

### Measures

#### Averages of residues

We compare protein surfaces with respect to structural and hydropathic properties, reflected in different selection criteria and averaged over families or the entire data sets.

The mean fraction of residues having property *s* with respect to a reference property *r* in a family is:

$$\begin{array}{c} {\hat{f}}_{s|r}=\frac{1}{\left|C\right|}\sum_{G\in C}\frac{\left|G_{s}\cap G_{r}\right|}{\left|G_{r}\right|} \end{array}$$ (1)

where *C* is the set of proteins in a family, *G* is a protein, and, *G*_*s*_ is the set of residues on a protein meeting criteria *s*. Vertical bars denote size of sets. We will specify *s* and *r* according to the definitions above, using intersect notation to combine criteria when necessary. ${\hat{f}}_{\text{hydrophobe}|\text{protrusion}\cap \text{helix}}$, for instance, should be interpreted as the mean fraction of hydrophobes out of all protruding amino acids that are in helices.

We estimate weighted data set counts of amino acids with property *s* as:

$$\begin{array}{c} {\hat{N}}_{s}=\sum_{C\in D}\left(\frac{1}{\left|C\right|}\sum_{G\in C}\left|G_{s}\right|\right) \end{array}$$ (2)

where *D* is a data set, such as the set of peripheral proteins or the reference set. Similarly we quantify the weighted count of proteins that have at least one amino acid with property *s* as:

$$\begin{array}{c} {\hat{M}}_{s}=\sum_{C\in D}\left(\frac{1}{\left|C\right|}\sum_{G\in C}\text{H}\left(\left|G_{s}\right|\right)\right) \end{array}$$ (3)

where H is the Heaviside step function. Given a property *s* and reference property *r*, we estimate the weighted fraction in a data set, ${\hat{F}}_{s|r}$:

$$\begin{array}{c} {\hat{F}}_{s|r}=\frac{{\hat{N}}_{s\cap r}}{{\hat{N}}_{r}} \end{array}$$ (4)

or the weighted fraction of proteins that have at least one residue with the given property *s*:

$$\begin{array}{c} {\hat{E}}_{s}=\frac{{\hat{M}}_{s}}{\left|D\right|} \end{array}$$ (5)

With |*D*| being the number of families in the data set. When such fractions (Eqs 4 or 5) are reported, we estimate 95%-confidence intervals using a normal approximation to the binomial distribution, with |*D*| the total number of trials (Eq 5), or ${\hat{N}}_{r}$ serving as a real-number analog to the total number of trials (Eq 4).

#### Averages of co-insertable pairs

To analyse co-insertable residues, we estimate weighted data set counts of co-insertable pairs of residues with property *s*, as:

$$\begin{array}{c} {\hat{N}}_{s}^{\text{pair}}=\sum_{C\in D}\left(\frac{1}{\left|C\right|}\sum_{G\in C}\left|G_{s}^{\text{pair}}\right|\right) \end{array}$$ (6)

where $\left|G_{s}^{\text{pair}}\right|$ are the number of co-insertable amino acids pairs with property *s*. For quantification of the weighted count of proteins that have at least one co-insertable pair with property *s*, we calculate:

$$\begin{array}{c} {\hat{M}}_{s}^{\text{pair}}=\sum_{C\in D}\left(\frac{1}{\left|C\right|}\sum_{G\in C}H\left(\left|G_{s}^{\text{pair}}\right|\right)\right) \end{array}$$ (7)

Considering the set of co-insertable amino acid pairs in a protein, *G*^pair^, we will denote the set of pairs where at least one of the amino acids is a protruding hydrophobe as $G_{\text{one}}^{\text{pair}}$, and the set where both are protruding hydrophobes as $G_{\text{both}}^{\text{pair}}$. We will report the weighted fraction of proteins that have co-insertable protruding hydrophobes as:

$$\begin{array}{c} {\hat{E}}_{\text{both}}^{\text{pair}}=\frac{{\hat{M}}_{\text{both}}^{\text{pair}}}{\left|D\right|} \end{array}$$ (8)

and the weighted frequency of co-insertion of protruding hydrophobes as:

$$\begin{array}{c} {\hat{F}}_{\text{both}|\text{one}}^{\text{pair}}=\frac{{\hat{N}}_{\text{both}}^{\text{pair}}}{{\hat{N}}_{\text{one}}^{\text{pair}}} \end{array}$$ (9)

Note that ${\hat{F}}_{\text{both}|\text{one}}^{\text{pair}}$ estimates the conditional probability that both amino acids of a co-insertable pair are protruding hydrophobes, given that one of them is. The tendency for protruding hydrophobes to be located at co-insertable positions can then be quantified by comparing with a null model for each set. We obtain these null models by randomly reassigning the hydrophobic amino acids to other protruding locations in the same protein.

#### Comparison between data sets

The frequency of properties in different data sets, are compared via weighted fractions. For two data sets, *A* and *B*, we compare a certain weighted fraction $\hat{F}$ using the odds ratio, $R(A,B,\hat{F})$:

$$\begin{array}{c} R\left(A,B,\hat{F}\right)=\frac{{\hat{F}}^{A}(1-{\hat{F}}^{B})}{{\hat{F}}^{B}(1-{\hat{F}}^{A})} \end{array}$$ (10)

where ${\hat{F}}^{A}$ denotes the fraction ${\hat{F}}_{s|r}$ obtained for data set *A*. We will report ln R, which is symmetric around 0, so that $\text{ln}\;R(A,B,\hat{F})=-\text{ln}\;R(B,A,\hat{F})$. Wald 95%-confidence intervals for ln R are calculated with ${\hat{N}}_{s\cap r}$ and $\left({\hat{N}}_{r}-{\hat{N}}_{s\cap r}\right)$ serving as real number analogs for the count of successes and failures in the data sets compared. When ${\hat{F}}_{\text{both}|\text{one}}^{\text{pair}}$ is compared, the corresponding counts of successes and failures are ${\hat{N}}_{\text{both}}^{\text{pair}}$ and ${\hat{N}}_{\text{both}}^{\text{pair}}-{\hat{N}}_{\text{one}}^{\text{pair}}$, respectively.

#### Comparison of experimentally verified and predicted binding sites

We define two vectors which we then compare to evaluate the distance between experimentally verified and predicted membrane binding residues. The C_α_-coordinate of experimentally verified membrane binding residues functions as a proxy for the membrane, and the vector defined by the latter residues and the center of mass (COM) of the protein is used as a reference to which we compare the vector defined by the protein COM and the Likely Inserted Hydrophobe. Given a set of identified or predicted membrane interacting residues, *I*, we compute the vector, **t**_*I*_:

$$\begin{array}{c} t_{I}=\frac{1}{\left|I\right|}\sum_{a\in I}V_{a}-\frac{1}{\left|G_{*}\right|}\sum_{a\in G_{*}}V_{a} \end{array}$$ (11)

where **V**_*a*_ denotes the C_α_-coordinates of residue *a*, and G_*_ is the set of all residues in the protein. We will denote vectors obtained for experimentally identified membrane binding residues as $t_{I_{e}}$, and those obtained for a Likely Inserted Hydrophobe as $t_{I_{p}}$. We then measure the angle $∠t_{I_{e}}t_{I_{p}}$ between the two vectors for each protein in the dataset of known binding sites.

### Implementation

The solvent accessible area was calculated with MMTK [74] (version 2.9.0), and the convex hull was calculated with Qhull [75] via scipy [76] (version 0.13.3). Proportion test confidence intervals were calculated with R [77] (Version 2.12.0), odds ratios and corresponding confidence intervals were calculated with the R-package epitools [78] (version 0.5-6). Secondary structure annotations were computed with the CMBI DSSP implementation [79] (version 2.0.4). For construction of the set ‘Peripheral-P’ and ‘Reference Proteins’ SCOPe version 2.06 was used. PISA predictions were obtained through the “Protein interfaces, surfaces and assemblies” service PISA at the European Bioinformatics Institute. (http://www.ebi.ac.uk/pdbe/prot_int/pistart.html). Where PISA predicted that the asymmetric unit represents the most stable quaternary structure in solution, we obtained structures from the Protein Data Bank (http://www.rcsb.org/) [33]. Otherwise the analyses were implemented by us, using Python and R. Plots were produced with R, and other visualisations using VMD (Visual Molecular Dynamics) [80]. Data sets of peripheral membrane proteins were generated on a snapshot of the OPM-database extracted the 23. Dec. 2013.

## Supporting information

S1 Text Supplementary tables and analysis. Analysis to assess the roboustness of some results to quaternary structure modelling, and specification of proteins and binding sites compared with experiment.
(PDF)

S1 Dataset The set ‘Peripheral’. Comma-separated file with PDB IDs for the set and the OPM classification of these at the time of analysis. The PDB IDs also serve to identify entries in the OPM database.
(CSV)

S2 Dataset The set ‘Non-binding surfaces’. Comma-separated file with PDB IDs for the set and the OPM classification of these at the time of analysis. The PDB IDs also serve to identify entries in the OPM database.
(CSV)

S3 Dataset The set ‘Peripheral-P’. PDB IDs for the set and the OPM classification of these at the time of analysis. The PDB IDs also serve to identify entries in the OPM database.
(CSV)

S4 Dataset The set ‘Reference Proteins’. PDB IDs for the set.
(CSV)

S5 Dataset Surface properties for all sets. Calculated properties for exposed residues in all sets. For the ‘Non-binding surfaces’ we have only included residues in the analysed fragments. PDB IDs, chain IDs and residue IDs refer to OPM quaternary structure models for the sets ‘Peripheral’ and ‘Non-binding surfaces’, and to the PISA generated quaternary structure models for the sets ‘Peripheral-P’ and ‘Reference Proteins’. The local density parameter *d* is identified as ‘local density’, the number of hydrophobes co-insertable to a residue is identified as ‘co insertables’, the solvent accessible surface area of a side chain ((*nm*)^2^) is identified as ‘sidechain sasa’, the ‘Likely inserted hydrophobe’ is identified as ‘LIH’, and other column names are self-explanatory.
(ZIP)
