# Supplementary material for: A model for hydrophobic protrusions on peripheral membrane proteins
Source: PLoS Comput Biol. 2018 Jul 26;14(7):e1006325. doi: 10.1371/journal.pcbi.1006325 (PMC6080788; doi:10.1371/journal.pcbi.1006325)
Supplement: S1 Text — Analysis to assess the roboustness of some results to quaternary structure modelling, and specification of proteins and binding sites compared with experiment. (PDF) [file pcbi.1006325.s001.pdf]

A model for hydrophobic protrusions on peripheral membrane proteins - Supporting information

Edvin Fuglebakk and Nathalie Reuter

PDB ID	interfacial binding site	LIH	co-insertables	angle	family
1RLW	F35, M38, L39, N95, Y96, V97, M98 [39]	**F35**	**M98 M38 L39**	19.2	C2-domain
1BYN	M173, G174, R233, F234, K235 [45, 46]	**M173**	**F234**	18.2	C2-domain
1UOV	V304, G305, I367, K369 [47]	L270		72.4	C2-domain
1GMI	I89, Y91 [48]	**I89**		6.8	C2-domain
1H6H	F35, Y94, V95 [40]	**Y94**	**F35**	23.7	PX domain
1CZS	W26, W27 [49]	**W26**	**W27** L79	4.3	Discodin domain
1D7P	M2199, F2200, L2251, L2252 [12]	**L2251**	**M2199 L2252 F2200**	18.9	Discodin domain
1T6M	W51, Y92, Y208, W246, Y250, Y252 [51, 50, 38]	**W246**	I47	8.5	Bacterial PLC
1H0A	L6, M10 [43]	I13	**L6**	20.4	ENTH domain
1LOX	L195 [52]	L71	Y292 F70 L192 **L195**	20.5	Lipoxygenases

Table A: Peripheral protein structures used for defining and parameterizing the model of hydrophobic protrusions. Family classifications are taken from OPM[25]. Although these proteins have not been included in Figure 7, we list here the *Likely inserted hydrophobe* (LIH), and protruding hydrophobes co-insertable with it. The *angle* column informs about the comparisons of orientations between our prediction and OPM (similar to those presented in Figure 7, as given by Eq 11).

S1 Data sets of proteins with experimentally verified membrane-binding sites

We used a small set of protein structures to establish the definition of protrusions and adjust the parameters c and n (Cf. *Materials and methods*). The dataset consists of structures of peripheral proteins with striking protrusions at their experimentally-verified membrane-binding site. Table A contains the list of PDB codes, the protein family to which they belong and the amino acids forming the membrane-binding site, along with some comparisons with predicted binding (*Likely inserted hydrophobes*) sites not presented in the manuscript.

We also collected a larger dataset of peripheral proteins with experimentally-identified binding sites. The structures and binding sites are listed in Table B, along with comparisons with our predictions (*Likely inserted hydrophobes*). This dataset does not overlap with the one listed in Table A and could thus be used for analysis purposes, and in particular for those results reported in Figure 7 of the main manuscript. This set has some overlap with the list provided by Lomize *et al.* [11].

The proteins presented in Figure 6 are taken from both of these sets. For convenience we have compiled a separate table, repeating the information about binding sites and comparisons with our predictions in Table C.

PDB ID	interfacial binding site	LIH	co-insertables	angle	classification
1DSY	M186 N189 R216 R249 R252 [53]	M269		166.8	C2-domain
1O7K	R43 I65 W80 [40]	L35		166.5	PX domain
1HYJ	V21 T22 [57]	W3		94.9	FYVE PIP_3 domain
1VFY	L185 L186 R193 [41]	**L186**	**L185**	14.7	FYVE PIP_3 domain
1PTR	L250 W252 L254 [42]	**L254**	M239	24.2	C1 domain
1A8A	T72 S144 W185 S228 S303 [55]	L29	**W185**	72.2	Annexins
1DM5	E142 S144 G145 [56]	L101	L260 I29 I185	49.2	Annexins
1IAZ	W112 W116 [58]	**W112**		6.3	Pore-forming Equinatoxin
1NB1	C1 G2 E4 T5 V6 G7 S18 W19 P20 V21 C22 G26 L27 P28 V29 [59]	**L27**	**W19**	77.4	Cyclotide
1POC	I2 K14 I78 [60]	L90	**I78** I1	71.2	Insect sec. PLA_2
1N28	V3 K10 L19 F23 F63 K115 [61]	**F63**		65.1	Vertebrate sec. PLA_2
1POA	W61 F64 Y110 [44]	W19	**F64** Y3	74.8	Vertebrate sec. PLA_2
1VAP	W20 W109 [62]	F3	M61 L19	90.0	Vertebrate sec. PLA_2
4P2P	W3 [67]	L19	**W3** M20	54.2	Vertebrate sec. PLA_2
1COY	M81 [63]	M332	L369 W333 Y437	28.5	GMC oxidoreductases
1PFO	W464 W466 [64]	L491	**W466** Y492 L462	14.4	Chol.-dep. Cytolysin
1D1H	W30 [65]	F6	**W30**	79.1	Spider toxins
1PXQ	W34 [66]	W34		0.0	Subtilosin A
2FNQ	W413 W449[68]	Y448	L514 **W449** F414 W413	12.6	Lipoxygenases
1G13	T90 L126 N136 [69]	W131	I162	51.0	ML domain
1EIN	P42 D96 T123 I252 [37]	**I252**	I255 I86 L93 L227	50.9	Fungal lipases
3PAK	Y164 R216 Y221 R222 [54]	L219		68.4	Lectin domain
1F6S	K98 V99 [23]				C-type lysozyme
2DA0	K18 K19 I23 K25 N30 N48 N77 [70]	**I23**		14.0	Pleckstrin-homology d.

Table B: Protein structures and corresponding membrane-binding sites used for systematic comparison with the *Likely Inserted Hydrophobe* (LIH). Protruding hydrophobes that are co-insertable with the LIH is listed in the column co-insertables. The *angle* column informs about the comparisons of orientations between our prediction and OPM (similar to those presented in Figure 7). Family classifications are from OPM [25], except for 3PAK and 1F6S which were taken from SCOPe [30] as the structures are not present in OPM. Quaternary structures are also taken from OPM, except for 3PAK and 1F6S; those were obtained from the literature. Residue numbering corresponds to that used in the listed PDB ID. All structures are either monomers or homo-oligomers where all chains are equally likely to interact with the membrane. Chain identifiers are therefore not provided.

PDB ID	interfacial binding site	LIH	co-insertables	angle	family
1RLW (A)	F35, M38, L39, N95, Y96, V97, M98 [39]	**F35**	**M98 M38 L39**	19.2	C2-domain
1H6H (B)	F35, Y94, V95 [40]	**Y94**	**F35**	23.7	PX domain
1POA (C)	W61 F64 Y110 [44]	W19	**F64** Y3	74.8	Vertebrate sec. PLA_2
1PTR (D)	L250 W252 L254 [42]	**L254**	M239	24.2	C1 domain
1H0A (E)	L6, M10 [43]	I13	**L6**	20.4	ENTH domain
1VFY (F)	L185 L186 R193 [41]	**L186**	**L185**	14.7	FYVE PIP_3 domain

Table C: Binding sites of common membrane-binding domains presented in Figure 6. All of these proteins are also listed in Tables A and B, and the table format is specified there. They are repeated here for ease of comparison with Figure 6, and the corresponding panel in that figure is indicated in parenthesis in the column PDB ID.

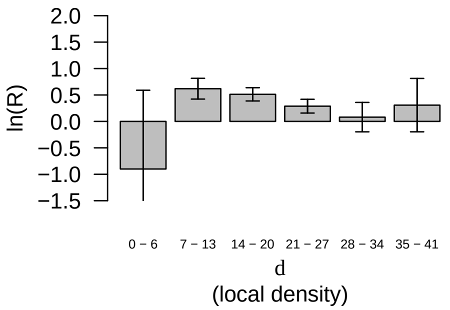

Figure A: The plot shows the logarithm of the odds-ratio comparing the frequency of hydrophobes on *vertex* residues in the set *Peripheral-P* and the *Reference proteins*. Positive values reflect higher frequencies in the peripheral proteins. See caption of corresponding Figure 3 in main text.

S2 Additional analysis performed

In the manuscript we have presented analysis on two pairs of data sets for analysis that aim to contrast surface properties between peripheral membrane proteins and other proteins. For analysis that aim to characterize protrusions on peripheral proteins, we have chosen to present these results only for one of the pairs. This is because we consider this a better representation of peripheral membrane binders, as quaternary structure has been more carefully scrutinized. Also, one of these analysis can only be done for the set *Peripheral*, namely the comparison with the OPM-database [25] presented in the manuscript (Figure 8). We present in this supplementary material(Figures A, B and C), analysis of the sets *Peripheral-P* and *Reference proteins*, corresponding to the analysis of the sets *Peripheral* and *Non-binding surfaces* presented in Figures 3, 10 and 9. The conclusions drawn from the primary datasets are supported by the analysis of *Peripheral-P* and *Reference proteins*. The relative importance of large aliphatic residues on protruding locations in peripheral proteins is reproduced (Figure B). There is still a stronger contrast between the data sets when the analysis is restricted to vertex residues of low protein density (Figure A). The analysis of secondary structure elements also yields a result similar to what was obtained for the primary datasets (Figure C).

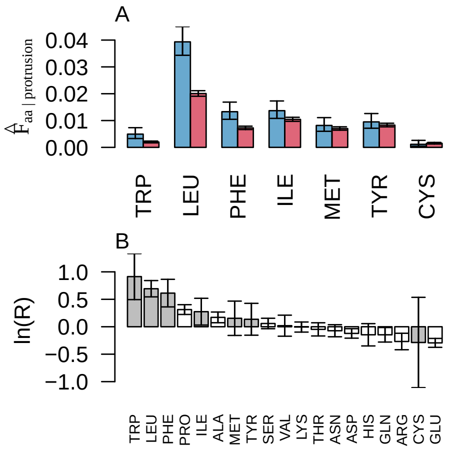

Figure B: Panel A shows the weighted fractions of hydrophobic amino acids on protrusions from the set *Peripheral-P* proteins (blue) and from proteins in the *Reference proteins* (red). In panel B, the contrast between the two sets is quantified by the odds ratio, so that positive values reflect higher frequencies in the set of peripheral proteins than in the reference set. See caption of corresponding Figure 10 in main text.

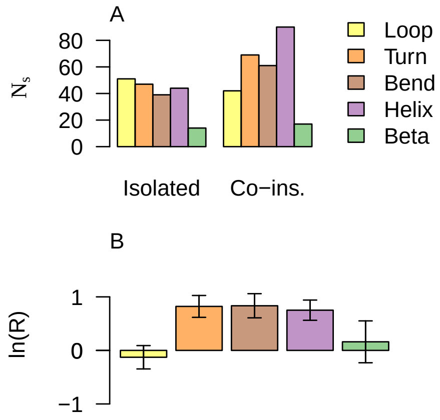

Figure C: Panel A shows the weighted number of *protruding hydrophobes* associated with the different types of secondary structure elements. We have differentiated between protrusions that have at least one co-insertable protruding hydrophobe (right, labeled “Co-ins.”), and those that have not (left, labeled “Isolated”). Panel B compares the weighted frequencies of hydrophobes on protruding secondary structures between the set *Peripheral-P* and the *Reference proteins*, using the odds ratio. Positive values reflect higher frequencies in the peripheral proteins.See caption of corresponding Figure 9 in main text.

References

See main text
